# Chemical characterization of the adhesive secretions of the salamander *Plethodon shermani* (Caudata, Plethodontidae)

**DOI:** 10.1038/s41598-017-05473-z

**Published:** 2017-07-27

**Authors:** Janek von Byern, Ingo Grunwald, Max Kosok, Ralph A. Saporito, Ursula Dicke, Oliver Wetjen, Karsten Thiel, Kai Borcherding, Thomas Kowalik, Martina Marchetti-Deschmann

**Affiliations:** 1 Ludwig Boltzmann Institute for Experimental and Clinical Traumatology, Austrian Cluster for Tissue Regeneration, Donaueschingenstrasse 13, 1200 Vienna, Austria; 2 University of Vienna, Faculty of Life Science, Core Facility Cell Imaging and Ultrastructure Research, Althanstrasse 14, 1090 Vienna, Austria; 3 Fraunhofer Institute for Manufacturing Technology and Advanced Materials (IFAM), Department of Adhesive Bonding Technology and Surfaces, Adhesives and Polymer Chemistry, Wiener Straße 12, 28359 Bremen, Germany; 4 Vienna University of Technology, Institute of Chemical Technologies and Analytics, Karlsplatz 13, 1040 Vienna, Austria; 5 John Carroll University, Department of Biology, University Heights, Ohio, 44118 USA; 6 University of Bremen, Brain Research Institute, Department of Behavioral Physiology, Bibliothekstraße 1, 28359 Bremen, Germany

## Abstract

Salamanders have developed a wide variety of antipredator mechanisms, including tail autotomy, colour patterns, and noxious skin secretions. As an addition to these tactics, the red-legged salamander (*Plethodon shermani*) uses adhesive secretions as part of its defensive strategy. The high bonding strength, the fast-curing nature, and the composition of the biobased materials makes salamander adhesives interesting for practical applications in the medical sector. To understand the adhesive secretions of *P*. *shermani*, its components were chemically analysed by energy dispersive X-ray spectroscopy (EDX), inductively coupled plasma mass spectrometry (ICP-MS), amino acid analysis, and spectroscopy (ATR-IR, Raman). In addition, proteins were separated by gel-electrophoresis and selected spots were characterised by peptide mass fingerprinting. The salamander secretion contains a high amount of water and predominantly proteins (around 77% in the dry stage). The gel-electrophoresis and peptide mass fingerprint analyses revealed a *de novo* set of peptides/proteins, largely with a pI between 5.0 and 8.0 and a molecular mass distribution between 10 and 170 kDa. Only low homologies with other proteins present in known databases could be identified. The results indicate that the secretions of the salamander *Plethodon* clearly differ chemically from those shown for other glue-producing terrestrial or marine species and thus represent a unique glue system.

## Introduction

To date, research on adhesives in nature has been primarily carried out on marine animals^1, 2^. Studies on glue synthesis and composition in terrestrial animals such as amphibians are rare^3–5^, although the bonding strength of their glue (up to 1.7 MPa for the Australian frog genus *Notaden*) is amongst the highest in the animal kingdom and comparable with industrial super glues such as cyanoacrylates (1.7 MPa)^6^. When provoked by potential predators, the frog *Notaden bennetti* secretes a sticky nontoxic material from its dorsal skin^5^. The secretions transform rapidly into an elastic solid (hydrogel) and adhere tightly to a wide range of materials, including glass, plastic, metal and even teflon^6^. Studies of the nature of these glues show that, in a dry state, the secretions contain few carbohydrates and consist mainly of proteins (13–400 kDa). Graham *et al*.^4^ indicate that the *Notaden* glue functions rather as a pressure-sensitive adhesive than through chemical mechanism such as that found in mussels and barnacles^7, 8^. This high bonding strength of the *Notaden* glue has already caused great interest for practical applications in the industrial^3, 9^ and medical sectors^10, 11^. This is a similar situation as has been given for the byssal system of mussels^12, 13^ or gecko nanotopography^14^.

Salamanders (caudates) have developed a wide variety of antipredator strategies^15^. The most effective strategies are various behavior patterns, tail autotomy, colour patterns, and the secretion of toxic, noxious or adhesive skin secretions^16–18^. The red–legged salamander *Plethodon shermani* is, besides *Ambystoma* spp., *Batrachoseps* spp. and *Bolitoglossa* spp., one of the few North American salamander species which secretes adhesive as an antipredator strategy^15, 19, 20^. Behavioural observations of the plethodontid salamander *Batrachoseps attenuatus* have demonstrated the effectiveness of this gluing strategy: Once attacked and grasped by a snake, the salamander loops its tail around the snake’s head and coats the snake with a sticky viscous fluid^19^. The adhesives in the secretion harden within seconds upon exposure to air^20, 21^ and immobilize the snake immediately^19^. The salamander then escapes, and the snake is unable to free itself for up to 48 hrs^19^.

Morphological and histochemical studies of *Plethodon shermani* have shown that the adhesive glands are mainly distributed along the lateral edges of the tail ridge and in the parotid region. Two gland types occur in the dermal layer of the skin^22, 23^: The mucous glands are distributed all over the skin and their secretory cells are densely packed with flocculent material, mostly acidic glycoproteins (positive staining for Alcian blue around pH 2.5 as well as PAS^23^. The granular glands are present in all skin regions, but are especially concentrated and larger in the parotoid area of the head and in the tail. The granular glands secrete various granules of different sizes and densities that contain only basic proteinaceous material (strong staining with Biebrich scarlet at pH 6.0 and weakly at pH 8.5, but no reactivity at pH 9.5 and 10.5)^23^. In the isolated secretion, positive reactions for acidic and basic proteins are seen, indicating that both glands contribute to glue formation.

While much is known about the toxic skin secretions in salamanders^16, 24, 25^ and adhesives in frogs^6, 9^, only marginal data are currently available on the adhesive nature of salamander secretions. With this study, we aim to survey for the first time the secretions of the gluing species *Plethodon shermani* and to characterize the adhesive secretion in greater detail.

## Results

### Secretion Handling

The exposed salamander secretion sticks irreversibly within seconds to all types of material as given for the *Notaden* glue (see description above). We were unable to stop or temporarily delay its hardening process (e.g. in a vacuum or by cooling it down) or otherwise maintain the secretions in a liquid state. Therefore, we were only able to use the cured adhesive and dissolve it using various buffers.

### Weight lost

**Figure 1:**
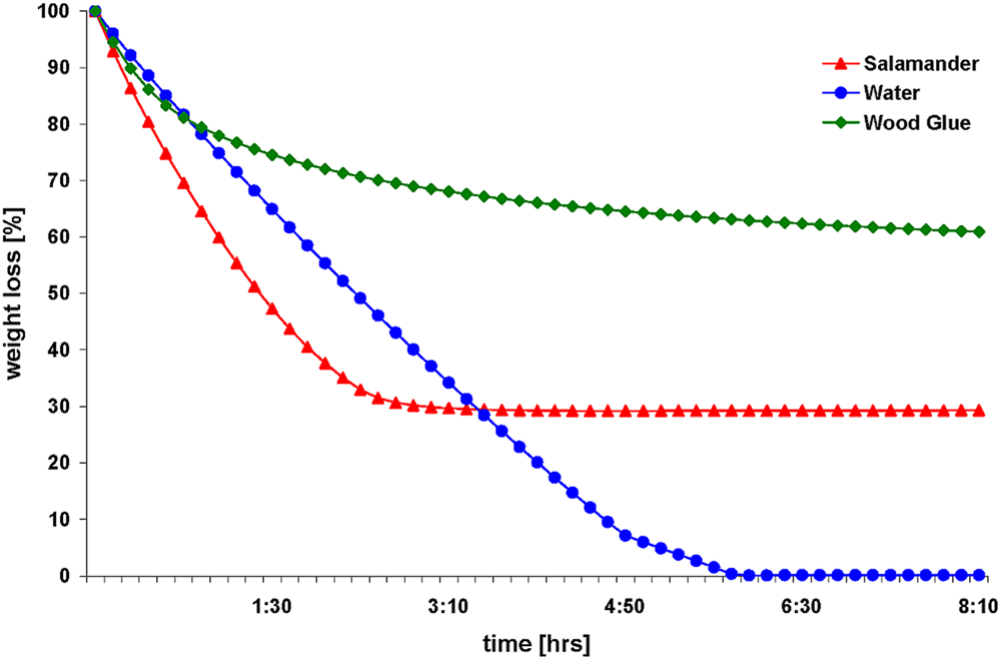
Drying of the salamander adhesive. Under low humid conditions (36% r. H., 24° temperature), the *Plethodon* secretion (red lines and triangles) shows a fast and high weight loss of 70% within 3 hrs in relation water alone (blue line and dots, final minima after 5 1/2 hrs). The water in the water-based wood glue (green line and squares), evaporates relatively slowly with a loss of only 30% of its weight and still reaches no stable endpoint after an 8-hr analysis.

The salamander secretion showed an increased rate of weight loss and reached its dry stage after 3 hrs, with a total weight loss of around 70% (Fig. 1). In contrast to this, tap water as control exhibited a continuous weight loss and needed longer (around 5 1/2 hrs) to evaporate completely. The aqueous wood glue, in contrast, evaporated much more slowly (no stable endpoint reached after 8 hrs) than water and the salamander secretion. It possesses an aqueous content of around 30%.

### Protein and carbohydrate quantification

Protein assay indicated that the salamander glue consists of 78% protein (w/w), while the colorimetric assay showed that the air-dried *Plethodon* glue contains only 0.41% carbohydrates (w/w). The glue dissolved more easily in the protein and carbohydrate assay buffers when heated up compared to a non-heated approach. The control measurement of bovine albumin serum was negative for the carbohydrate quantification.

### Elemental analysis

**Figure 2:**
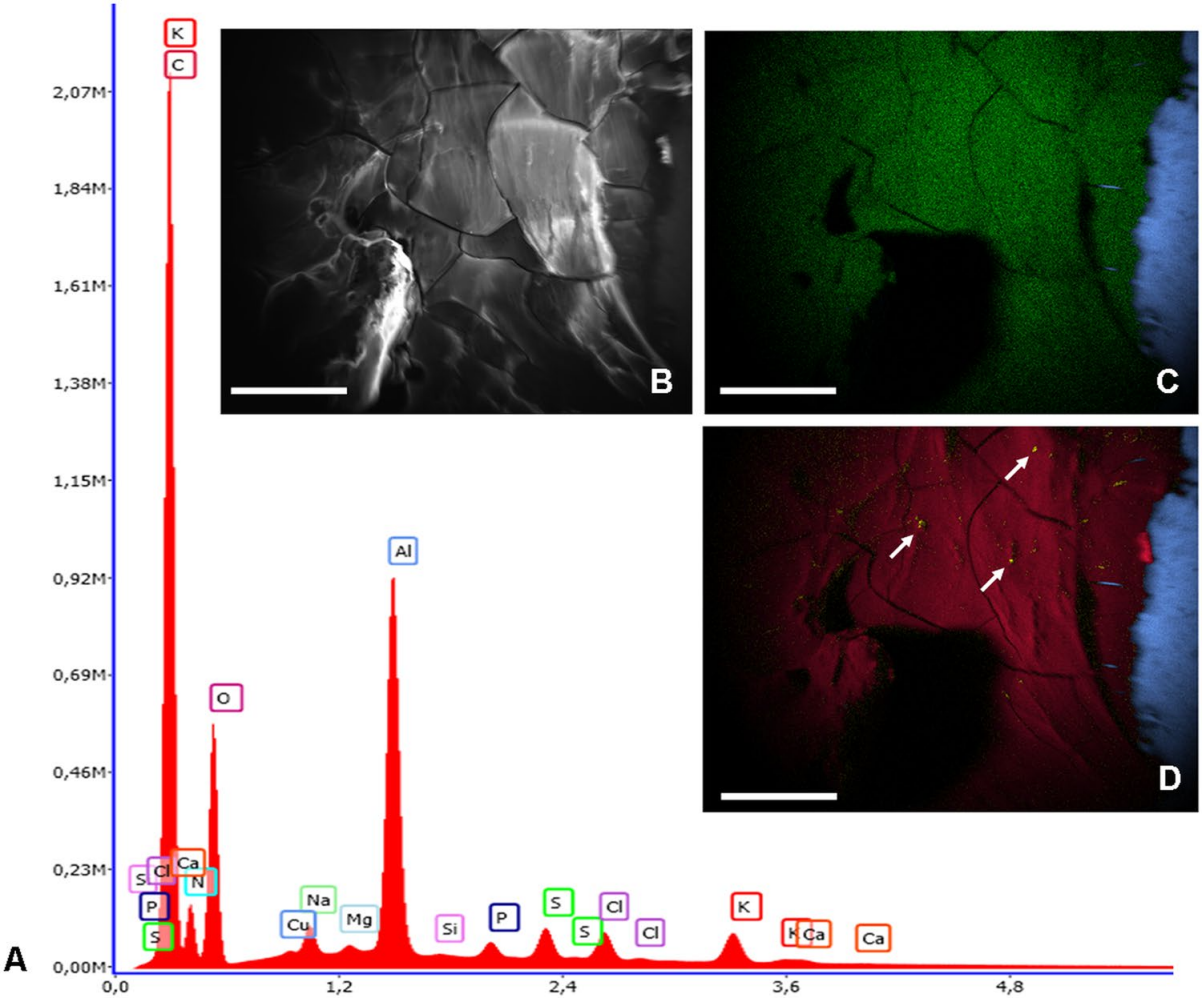
(**A**) Spectrum of the elemental composition of the salamander secretion. Insert (**B**) SEM and corresponding dot mappings for the elements (**C**) sulphur (green) and aluminium (blue) resp. (**D**) carbon (red), aluminium (blue) and calcium (yellow spots and white arrows).

The elemental analysis by EDX showed that the dried secretion mainly consists of carbon (≈60 at.%), nitrogen (≈16 at.%), oxygen (≈18 at.%) with a smaller amount of sodium (≈0.45 at.%), potassium (≈0.29 at.%), sulphur (≈0.24 at.%), chloride (≈0.22 at.%) and phosphorus (≈0.12 at.%) (Fig. 2). Other elements such as magnesium and calcium were below detection limits (<0.1 at.%), however dot mapping of the sample indicates the presence of local spots for calcium (Fig. 2). The blank SEM stubs consist of aluminium and copper only.

Measurement by ICP-MS confirmed the presence of potassium (13.000 ppm) and sodium (1.000 ppm) in the salamander glue; furthermore, calcium (2.000 ppm), magnesium (500 ppm), iron (200 ppm), manganese (29 ppm), copper (23 ppm) and zinc (29 ppm) in addition to rubidium, strontium, barium and lead at concentrations below 20 ppm could be confirmed via this more sensitive approach. Surprisingly, a concentration of arsenic (200 ppm) could be measured within the salamander secretion. The presence of other elements, such as Al, Li, Be, V, Cr, Co, Ni, Sr, Mo and Cd in the salamander adhesive, could not be confirmed by ICP-MS measurement; these elements were below the detection limit of the measurement procedure. Due to analytical limitations, sulphur and chlorine were not part of the element screening analysis using the selected ICP-MS equipment.

### Amino acid analysis

The amino acid analyses showed that the total protein content of the dried secretion was at least 78%. Eighteen amino acids were identified by hydrolysis (Table 1). The amino acid glycine was the most prominent of these, with a value of 7.8%. The sample contained more polar (52%) than non-polar (48%) and hydrophobic (38%) amino acids. Moreover, slightly more amino acids with acidic side chains (AsX and GlX in total 21%) than with basic ones (His, Lys, Arg in total about 12%) were present. Furthermore, a low level of L-3,4-Dihydroxyphenylalanin (L-DOPA) (0.12%) could be measured in the salamander adhesive.

**Table 1:** Amino acid composition (values in residues per hundred) of the *Plethodon shermani* secretions (present study) with previous data given for *Notaden bennetti* ^5, 35^, *Euperipatoides kanangrensis* ^36^, *Asterias rubens* ^64^ and *Holothuria forskali* ^39^, *Dosima fascicularis* ^37^ and *Lepas anatifera* ^43^, *Megabalanus rosa* ^42^ and *Balanus crenatus* ^65^ as well as *Phragmatopoma californica* ^47^. N.﻿D. = not determined.

Amino acid	Side chains		*Plethodon*	*Notaden*	*Euperi-patoides*	*Asterias*	*Holothuria*	*Dosima*	*Lepas*	*Mega-balanus*	*Balanus*	*Phragmato-poma*
AsX	Polar	Negative	7.9	7.2	5.0	11.8	7.8	9.3	8.8	9.1	7.8	2.9
Thr	Polar		5.0	4.4	3.6	7.8	8.7	4.8	5.3	7.0	6.6	2.2
Ser	Polar		4.3	3.8	4.2	7.6	6.0	6.6	8.8	9.9	11.4	28.5
GlX	Polar	Negative	8.3	14.1	5.1	10.2	9.1	10.3	9.1	9.1	9.1	1.4
Gly	Non-polar		7.8	15.8	27.3	9.7	26.6	4.2	9.6	7.9	8.3	26.2
Ala	Non-polar	Hydrophobic	3.2	2.8	3.5	6.2	8.8	5.6	9.0	7.5	6.9	9.8
Val	Non-polar	Hydrophobic	5.7	6.2	5.2	6.7	3.8	6.1	6.7	7.3	2.7	3.4
Met	Non-polar	Hydrophobic	1.3	1.1	1.4	1.7	1.0	0.5	0.6	1.6	0.7	0.0
Ile	Non-polar	Hydrophobic	4.2	4.8	5.1	4.5	2.8	5.7	5.9	5.3	4.4	0.6
Leu	Non-polar	Hydrophobic	5.6	6.9	4.5	6.1	3.7	8.1	9.8	8.3	8.8	3.4
Tyr	Polar		4.5	2.2	3.8	2.7	2.0	2.7	3.7	4.2	4.9	4.0
Phe	Non-polar	Hydrophobic	4.1	3.8	2.8	3.8	2.0	5.1	3.7	3.7	3.7	1.1
His	Positive	Polar	1.6	3.1	2.2	5.6	2.6	0.4	2.7	1.3	2.3	3.5
Lys	Positive	Polar	5.1	5.8	6.9	2.1	3.1	2.3	3.9	5.7	5.5	4.4
Arg	Positive	Polar	2.8	3.8	3.9	4.1	5.0	6.4	6.1	5.6	5.9	2.9
Pro	Non-polar	Hydrophobic	4.2	8.8	13.1	6.1	5.5	4.3	5.0	4.9	8.4	2.7
Cys/2	Polar		0.5	0.7	0.0	3.2	1.4	0.7	1.1	1.6	6.8	0.4
Trp	Non-polar	Hydrophobic	1.4	N.D.	N.D.	N.D.	N.D.	0.1	0.6	0.0	0.0	N.D.
Hyp	Others		N.D.	3.0	3.0	N.D.	0.0	N.D.	N.D.	N.D.	N.D.	N.D.
L-DOPA	Others		0.1	N.D.	N.D.	N.D.	N.D.	0.0	N.D.	N.D.	N.D.	2.1
Negative [%]			21	22	10	22	17	24	18	18	16	4
Polar [%]			52	46	34	55	46	52	49	54	58	50
Non-polar [%]			48	51	63	45	54	48	51	46	42	47
Hydrophobic [%]			38	35	35	35	28	43	41	39	34	21
Positive [%]			12	13	13	12	11	11	13	13	13	11
Others [%]			0	3	3	N.D.	0	0	N.D.	N.D.	N.D.	2
Water content [%]			70	85–90	90	N.D.	N.D.	92	N.D.	N.D.	N.D.	N.D.
Protein content [% dry weight]			78	55–60	55	20.6	59	84	N.D.	90	84	N.D.
Sugar content [% dry weight]			0.41	0.75	1.3	8	39	1.5	N.D.	N.D.	1.05	N.D.

### ATR-IR spectroscopy

**Figure 3:**
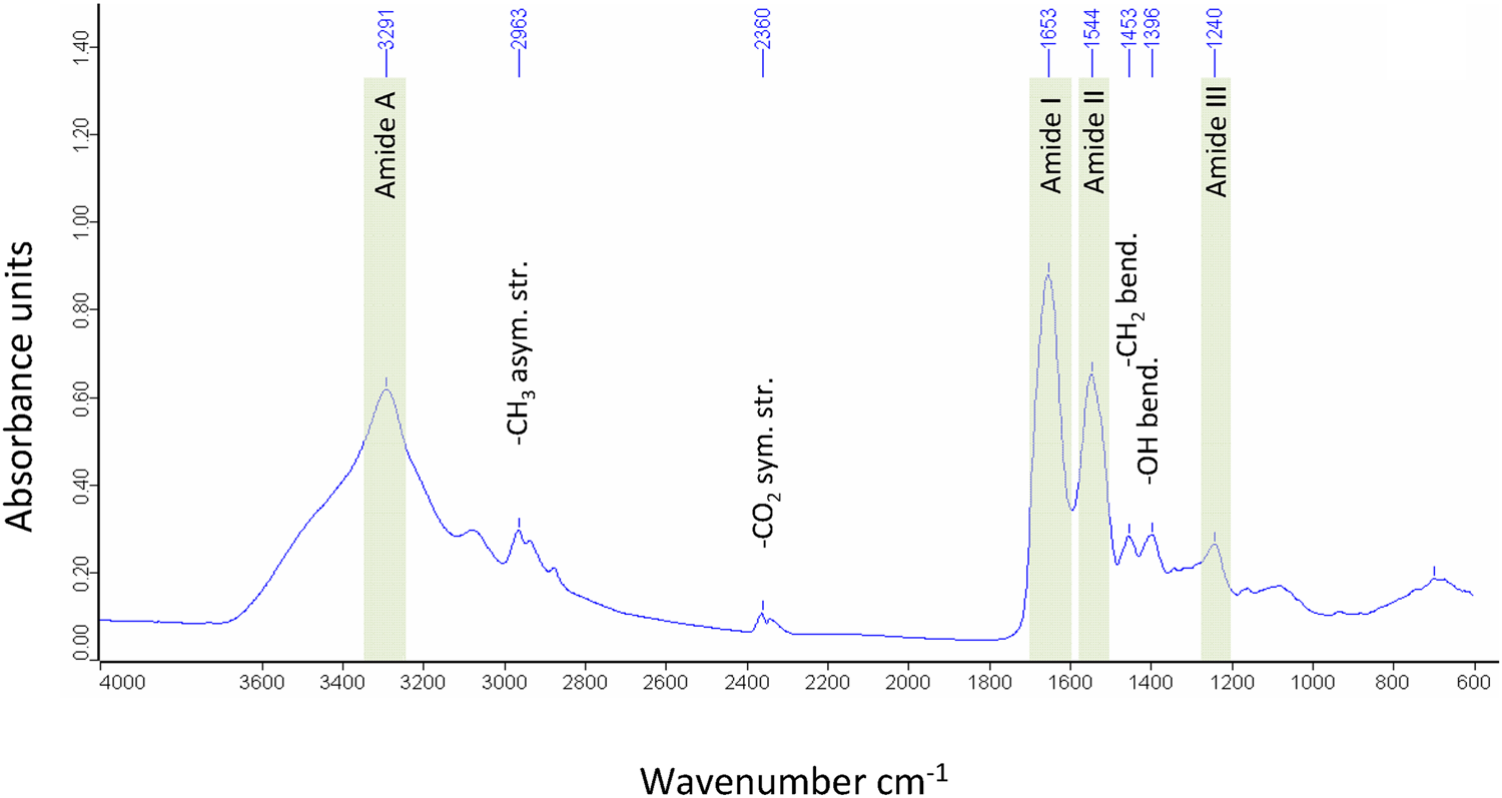
FTIR spectrum of dry salamander secretion. The region between 4.000 and 600 cm^−1^ is magnified and prominant vibrations from amide bonds have been colored green.

In freshly secreted samples, a strong signal from absorbed water molecules (O-H stretching region 3000–3650 cm^−1^; H-O-H- bending region 1600–1700 cm^−1^) could be detected (Fig. 3). Drying at +80 °C resulted in a significantly better spectrum corresponding to proteins (amide I at 1656 cm^−1^)^26^. Distinct peaks at 3291 cm^−1^ and 1545 cm^−1^ shown in the spectrum of the dried secretion indicated the absorption of an amide II vibration characteristic for N-H stretching^26^ (1580–1510 cm^−1^)^27^. The spectral peaks 1452 сm^−1^ and 1395 сm^−1^ were characterized by vibrations of the C-H bond in methyl and methylene groups of peptide fragments^28^. The analysed proteinaceous salamander secretion did not contain detectable amounts of phosphate (1253 cm^−1^)^29^ or polysaccharides (1150–1000 cm^−1^)^30^.

### Raman spectroscopy

The Raman data resembled a spectrum of a biological adhesive material composed mostly of proteinaceous material. Peaks between 2800 cm^−1^ and 3000 cm^−1^ were from -C-H bonds, e.g. found for I –CH_2_ symmetric and –CH_3_ stretching vibrations of organic molecules from biological samples.

**Figure 4:**
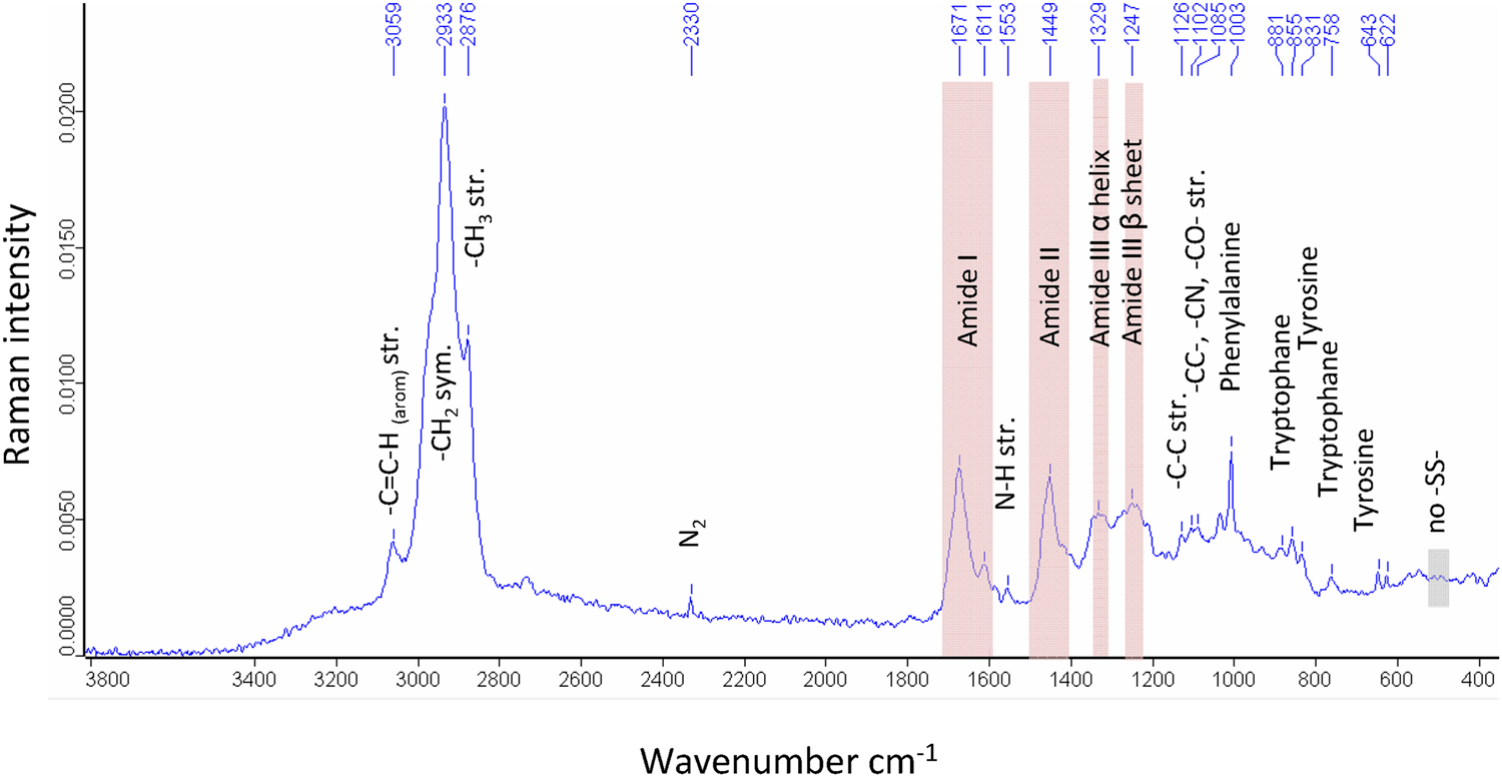
Raman spectrum of the dry salamander secretion. The region between 3.800 and 380 cm^−1^ is magnified and prominant vibrations from amide bonds have been colored red. Addtional vibrations, e.g. typical for amino acids, have been added to the spectrum.

A clear signal could be detected for the Amide I (between 1610 cm^−^ to 1690 cm^−1^) and Amide II (1420 cm^−1^ to 1480 cm^−1^) bands. The Amide III bands showed weaker signals that could be divided into α-helix (1310 cm^−1^ to 1330 cm^−1^) and β-sheet (1235 cm^−1^ to 1250 cm^−1^) signals^31^. Taken together, the results confirmed a relatively higher content of β-sheet structure than β-turn, α-helix and random coil (1680–1665 cm^−1^)^31^ (Fig. 4). In addition, a strong signal for phenylalanine (1003 cm^−1^ and 1606 cm^−1^)^31, 32^ bands arising from tyrosine residues (855 and 832 cm^−1^)^32^ and tryptophan could be detected.

Vibrations for L-DOPA (735–730 cm^−1^)^33^ were not found and may correlate with the low amount found in the amino acid analyses. Furthermore, strong vibrations from disulphide bridges (510–540 cm^−1^)^32^ could also not be detected in the salamander secretion. Components from DNA nucleotide base-ring vibrations like guanine (665 cm^−1^ and 1575 cm^−1^), cytosine and uracil (785 cm^−1^) and adenine (720 cm^−1^) were also absent in detectable amounts^29^.

### Alkaloid analysis

GC-MS analysis of the *P*. *shermani* skin extract yielded no detectable skin alkaloids.

### Sequence analysis

**Figure 5:**
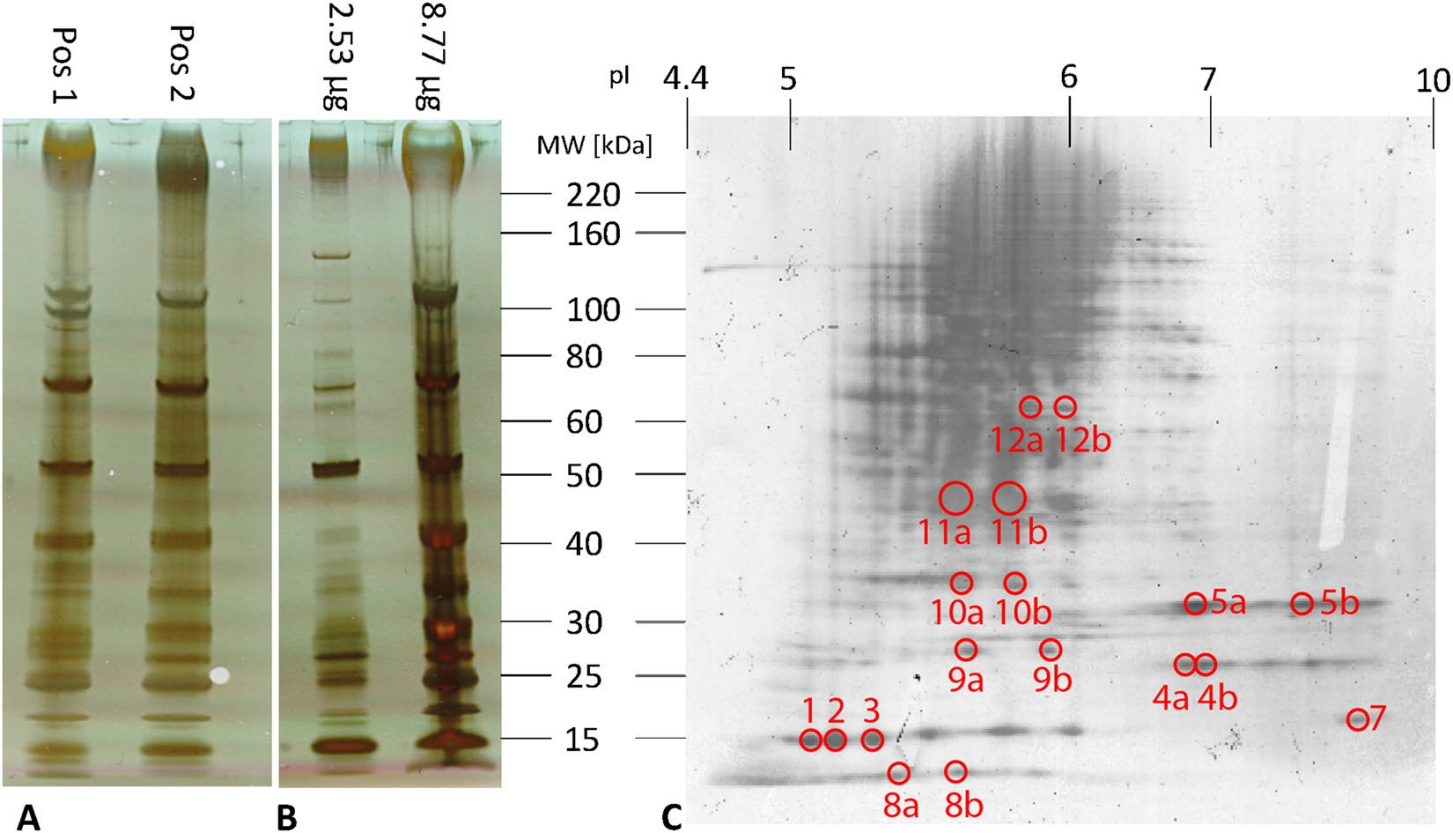
1D- and 2D-PAGE. 1D-PAGE of the salamander secretion (**A**) extracted with LDS buffer from two different positions of an Aclar® foil (approx. 0.75 cm^2^ each) and (**B**) different concentrations of bioadhesive scratched from the Aclar® foil over an area of 2 cm^2^ solubilized in LDS buffer. (**C**) 2D-PAGE of 44 µg protein extracted from a salamander bioadhesive.

In 1D gel electrophoresis (**1D-PAGE**), two intensive bands at approx. 50 and 70 kDa were observed, the first one most likely being a double band. In total, about 18 protein bands with a molecular mass ranging from 10 to 160 kDa were detected. Interestingly, the two samples taken from two different spots on the Aclar® foil (sample type Pos 1, Pos 2) showed similar yet non-identical protein patterns. The double band at 50 kDa showed a lower intensity for the lower band and one band at approx. 100 kDa band was missing for the sample taken at Pos 1 (Fig. 5a). This can be explained by the fact that the secreted bioadhesive is expected to have a certain degree of biological variation caused by the different stress levels of the animals.

Separating the sample containing the protein extract from the isolated glue scratched from the Aclar® foil (sample type “Mix”) showed a similar, but again different, protein pattern (Fig. 5b). The protein band at 70 kDa was significantly less intensive, the protein band at 50 kDa was the most intensive band and the proteins between 30 and 50 kDa were almost not extracted. However, an additional protein band below 70 kDa was observed. This difference can be explained by the different extraction efficiency of the buffer systems used and by the biological variation of the secreted proteins. Pos 1 and Pos 2 were extracted with a buffer with a high solubility strength (LDS) for SDS-PAGE, while the Mix was extracted with urea, thiourea and CHAPS in preparation for 2D-PAGE. Furthermore, Pos 1 and 2 were extracted under denaturating conditions (DTT), allowing proteins rich in disulphide bridges to become more soluble. However, amino acid analysis revealed only a low amount of Cys, while Raman spectroscopy lacked an abundant signal for disulphide bridges, making this a less likely possibility. Without taking the biological variation in account, it can be assumed that proteins observed in the Mix are more easily dissolved in aqueous solvents, especially the protein at 70 kDa.

**2D-PAGE** of 44 µg protein extract shows that the protein pattern in this adhesive is rather complex. Prominent spots around 15, 25, and 30 kDa can be found in the 2D-PAGE (Fig. 5c), while the prominent protein bands at higher MW (50 to 170 kDa) led to insufficient protein separation due to sample complexity, and only a few spots are clearly observable. Most of the proteins were observed between pH 5.0 and 7.0.

While studying the presence of proteins in the secretions of a provoked animal, one has to keep in mind that the collected sample will represent mainly the proteome of the secreted glue but can also contain proteins coming from the animal’s skin, e.g. keratin. Although protein separation was hampered in the higher molecular mass range (>50 kDa), spots between 8 and 70 kDa were selected at different apparent MWs and pIs (Fig. 5c, Suppl. Table).

Comparable PMFs and identical fragmentation patterns for many peptides indicated that spots 1, 2 and 3 are protein isoforms (≈15 kDa, pI 5.2–5.5). The database search for the MS and MS/MS spectra did not give any significant results, but *de novo* sequencing in combination with a BLAST search resulted in a strong indication towards a NADH dehydrogenase subunit (subunit 2 or/and 4), a mitochondrial protein. Mitochondria are located where energy consumption is highest and since the adhesive collection was performed from syncytial gland cells, it is therefore conceivable that such proteins are present in the salamander secretion. The lower molecular mass (compared to the database entry) can be explained by protein degradation or sample preparation artefacts. Most peptides indicated subunit 4, but one peptide sequence showed similarity to NADH dehydrogenase subunit 2 in *Plethodon* sp. and subunit 6 in *Ambystoma* sp. Three peptides in spot 1 furthermore gave rise to the assumption that cytochrome b, also a mitochondrial protein, is present. However, one peptide had a higher similarity to a plethodontid modulating factor, which was also a result for the same peptide in spot 3. This finding has to be followed up to be better understood.

Also, in spots 9a and b (25 kDa, pI 5.8 and 6.0) peptide sequences had similarities to NADH dehydrogenase subunits (subunit 4 and 5), although one peptide also showed a partial, yet lower, sequence homology to cytochrome oxidase subunit 1 from *Plethodon*.

Also, gel spot 4a (≈25 kDa, pI 6.9) exhibited two peptides with sequences homologous to NADH dehydrogenase subunits from salamanders, one to subunit 4 and the other to subunit 5. Only one of them had a relevant correlation to proteins from *Plethodon*. However, gel spots 4a and 4b (≈25 kDa, pI 6.9 and 7.2) showed an unexpectedly high number of peptides that were identified with statistical significance from MS/MS spectra to be keratin from other vertebrates. Moreover, one peptide (m/z 1450.7) gave no identification after the MS/MS search, but *de novo* sequencing and the BLAST search indicated a partial sequence of keratin from *Ambystoma mexicanum*. It could be assumed that the presence of keratin in the secretion is the result of sample preparation as the glue was collected from the animal’s skin, where keratin is present^34^. Interestingly m/z 1400.7 gave a significant MS/MS search result in SwissProt (lysozyme from *Gorilla gorilla*), yet we have reasonable doubts and consider this to be a false positive.

Spots 5a and 5b have slightly higher MWs (≈30 kDa) and similar pIs (7.1 and 8.5 resp.). Four peptides showed identical fragmentation patterns and molecular masses for both gel spots, once more indicating protein isoforms. *De novo* sequencing furthermore corroborated that these protein isoforms are NADH dehydrogenase subunits. This assumption was substantiated by a significant MS/MS database search result for m/z 2659.4 (NCBI), identifying this peptide to be from the NADH dehydrogenase subunit 2 (cross species identification). Interestingly, 10a and b (≈35 kDa, pI 5.8 and 5.9) are also isoforms showing the same PMF and identical fragmentation pattern for three peptides. Although 10b gave a significant protein identification by PMF (centrin-3), this identification could not be confirmed by MS/MS (m/z 1400.7, 1421.7 and 1747.9). However, both sequence tags hinted again at NADH dehydrogenase subunit 2, and m/z 1747.9 in particular seems to be a highly conserved sequence of that subunit (all 166 BLAST search results, taxonomy: *Amphibia*).

Spot 7 (17 kDa, pI 9.3) did not give any clear results. Most of the peptide sequences were too short to provide any hints regarding protein identification. Only m/z 887.5 resulted in significant MS/MS identifications. The result from SwissProt was at the threshold level, yet of statistical significance. However, the species from which this protein was identified was not closely related to amphibia. Nevertheless, the result from NCBI gave an extensive sequence homology for a peptide belonging to rhodopsin from *Ambystoma tigrinum*. Rhodopsin is a biological pigment found in the rods of the retina and is a G-protein-coupled receptor (GPCR). However, it could not be excluded that part of the highly pigmented skin of *Plethodon shermani* was scratched off during glue collection, resulting in this sequence homology.

Protein isoforms were also confirmed (identical PMF, MS/MS for selected peptides) for spot 11a/b (≈45 kDa, pI 5.8 and 5.9) and 12 a/b (≈60 kDa, pI 6.0 and 6.1). These spots also had many peptides in common (including MS/MS information), giving rise to the assumption that 11a/b are degradation products of 12a/b or that 12a/b are multimers of 11a/b. The identification of spot 11a as a serine/threonine-protein phosphatase (cross-species identification with *Xenopus laevis*) was not confirmed by MS/MS (m/z 607.3 and 1749.9). De novo sequencing and the BLAST search did not give conclusive results.

Spots 8a and b (both having MWs < 10 kDa, pI 5.6 and 5.8) were not identified due to low molecular mass and insignificant mass spectra.

## Discussion

Although the composition of the skin secretions in *Plethodon shermani* has been previously studied^22, 23^ on a histochemical level, we still know little about the composition of the adhesive.

The present study indicate that the native secretion of *Plethodon* contains a relatively large amount of water (≈70%). A water content of 85–90% has also been measured in the adhesive skin secretions of the Australian frog *Notaden* and in the prey capture glue of the onychophoran species *Euperipatoides* ^35, 36^. Values beyond 90% have even been demonstrated in other species, i.e. 92% for the sponge-like cement of the marine goose barnacle *Dosima* ^37^ and 98% for the prey capture threads of the New Zealand glowworm larvae *Arachnocampa luminosa* ^38^. The water evaporation and thereby the hardening of the secretion in *Plethodon* appears to be faster compared to either water alone or a water-based wood adhesive. As the adhesive secretions in salamander are used for defence, i.e. to immobilize predators or coat their teeth with the secretion^19^, a fast-curing system is obligatory to ensure survival. In the capture system of *Arachnocampa*, it takes three days for the water to evaporate completely and the weight loss to be completed^38^. As this particular adhesive is used for prey capture under highly humid conditions^38^, a complete dehydration and loss of adhesiveness is thus prevented for as long as possible. Unfortunately, no data are available on the dehydration and curing speed for *Notaden* or any other glue-producing animal, which would indicate a general tendency of water evaporation speed in relation to the application of the adhesives by the animal.

The *Plethodon* secretions possess a high protein content (around 78% of dry weight according to Bradford assay), which could also be confirmed by the distinct amide peaks in the ATR-IT and Raman spectra. The histochemical characterization of the adhesive secretions indicates the presence of acidic proteins (pH 2.5–4.3; weak reaction at pH 1.0) and basic proteins (pH 6.0–8.5, no reactivity at pH 9.5 and 10.5)^23^. This staining result could also be observed in the 2D-PAGE, showing protein spots from pI 5.0 to pI 8.0, confirming that the contents of both glands were secreted during the stressing of the animal. At this moment, it remains unclear whether there are more or less proteins present compared to SDS-PAGE because of the chosen 2D-PAGE setup (7 cm gels, 3–10 NL and MW range). It is also possible that some of the proteins were not solubilized in the buffer system.

In relation to this, the glue of *Notaden* exhibits a lower protein amount (55–60% of dry weight), but most of the protein spots are in the same pI range (5.0–7.0), although some smaller proteins are very basic (pI 8.0–10.0)^35^. So far only one gland type, the granular gland, has been proposed as forming the adhesive secretions in *Notaden* ^9^, however a detailed morphological and histochemical characterization of the gland system is lacking. The absence of acidic secretions, like those produced by the mucous gland in *Plethodon*, in the *Notaden* glue may be a result of the different functions in both amphibians. *Notaden* uses the adhesive to attach to a partner during mating and fertilization^9^. *Notaden* only occasionally protect itself against ants and termites^5^, while *Plethodon* defends itself actively with its adhesive secretions. In other bioadhesives, proteinogenic contents lower than 20% (i.e. *Holothuria*, *Littorina)* ^39, 40^ or higher than 84% (i.e. *Lepas*, *Megabalanus*, *Dosima*)^37, 41–43^ have been measured, demonstrating a wide and diverse range.

Apart from the differences in the protein amount and pI range, variations regarding the molecular mass range in relation to other glue-producing species are also given for *Plethodon*. In an earlier study of *Plethodon*, a protein band with an approximate molecular mass of 70 kDa is shown in the SDS-Page image in the dorsal and ventral skin extract^22^. Furthermore, ventrally a band between 28.9 and 34.8 kDa occurs which is lacking in the dorsal extract^22^. In the isolated noxious/sticky secretion, distinct bands appear below 20 kDa, at 40 kDa and 120 kDa (apart from the ventral 28.9 to 34.8 kDa band), while the 70 kDa band is lacking in this sample type^22^. In addition to the results above^22^, a “dark protein band near the top of the gel (estimated 260–330 kDa from gels run longer)” (citation from^35^) could also be observed for the dorsal skin secretion of *﻿P*. *shermani* ^35^. The authors^35^ also refer to protein bands of 47 kDa and 100 kDA (“In addition to proteins of approximately 120, 100, 47, 27 and 14 kDa^22^, …”), however, these bands could not be observed by re-examining the study^22^.

In the present study, the secreted *Plethodon* glue from the dorsal and ventral skin area contain 18 prominent bands, mainly at approx. 15, 20, 25, 27, multiple bands at 30 and 35 kDa and further bands at 40, 50, 70, 100 and 120 kDa. Some of these bands correspond in their ranges to the dorsal/ventral skin extracts (28.9 to 34.8 kDa, 70 kDa) and sticky secretions (<20 kDa, 40 kDa, 120 kDa) of the previous study^22^.

It remains unclear whether proteins beyond 200 kDa are also present in the *Plethodon* secretions as observed earlier^35^. Since significant staining beyond 220 kDa is visible, providing an indication of high molecular weight compounds, respective gel electrophoretic analysis with agarose gels will be followed up in a further study. In *Notaden*, in contrast, there is a wider range (13–500 kDa), but with less prominent protein bands (>8) and a dominant glycoprotein (Nb-1R) at 350–500 kDa^4, 35^. Considering that only one gland type in *Notaden* is involved in glue production, it is interesting that there is a lower protein amount yet a wider mass range and high molecular proteins than in *Plethodon*. In addition to *Notaden*, the velvet worm *Euperipatoides* also possesses proteins with high (>600 kDa) and low molecular masses (13–19 kDa or lower than 13 kDa)^36^ and a dominant glycosylated protein (Er-P1) at 230/350 kDa^35^. In most other glue-producing species, however, the proteins are also smaller than 200 kDa: i.e. *Holothuria* = 220 kDa^39^; *Dosima* = 205.0 kDa^37^; *Littorina* = 118 kDa^44^; *Mytilus* = 110 kDa^45^ and *Megabalanus* = 100 kDa^7^.

There are also differences between the glue-producing species regarding their carbohydrate amounts. In some (i.e. *Holothuria*, *Littorina*) there is a high carbohydrate fraction (15–40%)^39, 40, 46^, while in most others (e.g. *Dosima*, *Lepas*, *Notaden*, *Euperipatoides*), the carbohydrate content is relatively low (<3% dry weight)^35, 37, 41^. In the *Plethodon* secretions, a low carbohydrate concentration (0.41%) could likewise be measured, although the secretory content of both gland types as well as the isolated secretion show a high affinity for sugar residues such as mannose (from the mucous gland) or L-fucose and N-acetyl-d-glucosamine (from the granular gland)^23^. However, respective sugar moieties could not be measured by ATR-IR and Raman, which is surely related to its low amount in the secretions compared to the protein fraction.

In view of the amino acid distribution, the skin secretions of *Plethodon* possess a similar range of negatively and positively charged amino acids compared to those shown for all other glue-producing animals (Table 1), while *Euperipatoides* and *Phragmatopoma* clearly exhibit lower values (10% and lower) for acidic amino acids^36, 47^. While the salamander secretion contains slightly more polar groups (e.g. Tyr) than other marine species (*Asterias*, *Dosima*, *Megabalanus*, *Balanus* and *Phragmatopoma*)^37, 42, 43, 47, 48^, the secretions of the terrestrial *Notaden* and *Euperipatoides* are rather unpolar. There are again similarities regarding the quantity of hydrophobic amino acids given for most animals, including *Plethodon*, and only the two goose-barnacles *Dosima* and *Lepas* appear to be clearly higher (>40%), while the sandcastle worm *Phragmatopoma* is lower (21%). In general, in most listed adhesives a clear dominance (values >10%) of a few amino acids in relation to others can be observed, e.g. Gly in *Notaden*, *Euperipatoides*, *Holothuria* and *Phragmatopoma*, GlX in *Notaden*, *Asterias*, *Dosima*, AsX in *Asterias* and *Dosima*, Ser in *Balanus* and *Phragmatopoma* or Pro in *Euperipatoides* (see Table 1 for details). In the adhesive secretions of the salamander *Plethodon*, in contrast, the composition is relative homogenous and all values are lower than 10%; there was no clear dominance of certain amino acids; nevertheless, also in *Plethodon* glue a slight tendency towards the amino acid Gly could be observed, similar as for other animals listed above. Although L-Dopa could be measured in the salamander glue (0.1%), its value is much lower in relation to those given for the sandcastle worm *Phragmatopoma*. Morphological studies furthermore have shown that DOPA-containing proteins could not be detected in the gland cells of *Plethodon shermani* ^23^, therefore an involvement of L-Dopa in glue formation seems to be unfavourable for the salamander.

Disulphide bonds are known to stabilise the protein complex and contribute to its cohesive strength^48^, and respective values of cysteine above 1% have been shown for the secretions of *Asterias*, *Holothuria*, *Lepas*, *Megabalanus* and *Balanus*. In the secretions of *Plethodon*, sulphur is present, as confirmed by EDX peaks and the amino acid methionine. Disulphide bonds, formed between the thiol groups of cysteine residues, could not be detected by Raman in the salamander secretions. Furthermore, the cysteine value is only around 0.5% and thus may not contribute as a main mechanism to the curing of the adhesive, as has been discussed for other biological adhesives^49^. In addition to the potential involvement in the stabilization of the adhesive secretion, this amino acid also serves as a pheromone for females^50^ or is part of the antioxidant protein pleurain-K1 (together with proline, free cysteine, tyrosine and tryptophan)^51^.

In addition to sulphur, in many marine adhesives, such as those from mussels^52^ and sandcastle worms^53^ as well as other species like caddisflies^54^, phosphorus also plays an important role in the phosphorylation of amino acids such as serine, threonine, tyrosine, histidine and aspartic acid^52^. Furthermore, divalent cations such as magnesium and calcium could be detected in adhesives^37, 47^ and to a lower extent also in the *Plethodon* glue by ICP-MS, supporting the hardening and stabilisation of the secretions. Although phosphoserine could not be chemically verified in the *Plethodon* adhesive, a phosphorylation of other potential available amino acids such as threonine, tyrosine, or aspartic acid could not be excluded at this stage.

Beyond the alkali metals and alkali earth metals also transitional metals such as iron, copper, manganese and zinc could be confirmed in *Plethodon*, as has been shown in the defence glue of the slug *Arion subfuscus* ^55^. However, while the iron, copper and manganese concentrations in the slug glue is much lower (around 2–7 ppm for all three elements) than those in the salamander glue (23 to 200 ppm), the opposite is given for zinc (70–340 ppm in the *Arion* ^55^, 29 ppm in the *Plethodon* glue). Arsenic (and to a much lower extent, the alkali metal rubidium and earth metals strontium and barium) could only be confirmed in the glue of *Plethodon* so far. Further investigations are planned to exclude a contamination through the habitat as has been shown for other amphibians^56^ or to confirm its presence in other glue-producing animals. Unfortunately, no element analysis is given for the secretions of the other two terrestrial animals *Notaden* and *Euperipatoides*.

Despite the high zinc content, the analyses of the *Arion* glue concluded that, in particular, an increase of the solubility of the slug glue, moreover of a specific iron-binding protein (15 kDa), could be confirmed^55^. In addition to slugs, iron is also known to take a central position as a Fe(DOPA)_3_ cross-link in mussel byssus formation^57^. Further studies in salamanders are planned to verify its involvement in glue function as given for the two mollusc groups.

In summary, as demonstrated earlier by Graham^35^, the adhesive secretions of the frog *Notaden* show strong similarities regarding their composition and protein sequence to those of the velvet worm *Euperipatoides*. In contrast, congruence within the amphibians could not be confirmed; the chemical differences between the frog *Notaden* and the salamander *Plethodon* are too large. Also, regarding the other glue-producing marine species, clear differences in the amino acid composition and protein size range were observed in *Plethodon*, confirming a completely unique glue composition.

## Material and Methods

Salamanders of the species *Plethodon shermani* were collected from populations in the vicinity of the Highlands Biological Station, North Carolina, USA. The collecting permit 10-SC00345 was issued by the North Carolina Wildlife Resources Commission Raleigh to Prof Dr Lynne D. Houck, Oregon State University, Cordley Hall, Oregon, USA. The wild-caught animals were housed in cooled boxes in a plastic container and transported according to ﻿A﻿merican and German animal welfare﻿ regulations via airplane to Ursula Dicke (University of Bremen). Directly before transport and immediately upon arrival at the airport in Germany, the animals were monitored by a veterinary officer. In Bremen, the animals were kept communally in terrariums with a substrate of moist soil and mulch, a temperature of 14 °C, a humidity of 80% and a dark-light cycle of 12 hrs. They were maintained on a diet of crickets. Collection of the salamander secretions followed the guidelines of the animal welfare law, approved by the Animal Care and Use Committee of the State Bremen, Germany.

**Figure 6:**
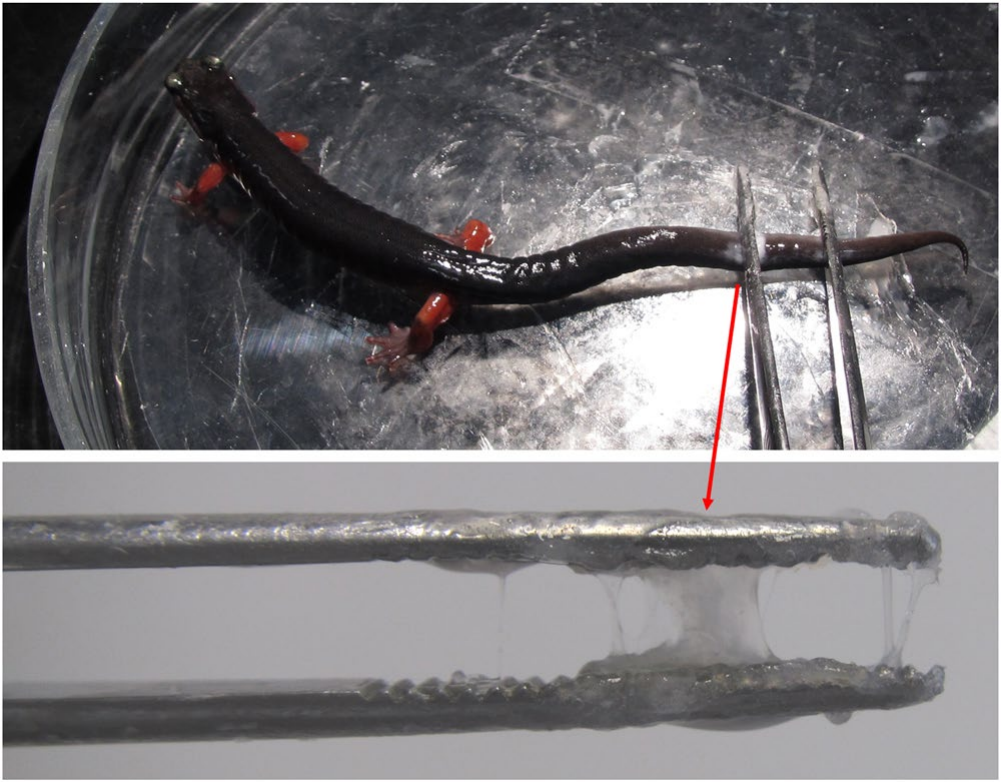
Isolated glue of *Plethodon shermani*, sticking to the metal forceps.

To collect the adhesive, the salamanders’ legs were gently twitched with forceps while the animal was kept in a large Petri dish. The provoked animals started to exude a visible amount of a milky mucous secretion. This liquid aggregated and bonded strongly within seconds to different surfaces (Petri dish glass, plastic foil and metal forceps) when exposed to air (Fig. 6). The adhesive was collected on clean Aclar® foils (Co. Groepl, Austria, Cat.-Nr. 10501-10), laid out in a petri dish and subsequently divided into aliquots for the analyses. Aclar® foil (made from fluorinated-chlorinated resins, see US Patent No. 5088603 A and US Patent No. 3256981 A) is chemically resistant to all used buffers and, more importantly, does not contaminate the sample because the glue does not have to be scratched from glass (silica contamination) or plastic (polymers, plasticizers, …). However, in the present study, secretions from the dorsal and ventral glands of one or several *P*. *shermani* were used for the following investigations as no morphological or histochemical differences could be observed in an earlier study^23^.

For the **weight loss experiments**, the freshly isolated salamander secretions (n = 3) were collected in pre-weighted Eppendorf tubes and dried at 24 °C (36% relative humidity) in a moisture analyser (Mod. MA35, Co. Sartorius, Germany). Continuous weight measurements were taken until no more weight loss was measurable. As a comparison, tap water and the aqueous wood glue Ponal^®^ (Henkel, Germany, based on polyvinyl acetate – PVA) (in the same weight range as for the *Plethodon* glue) were used in parallel and dried under the same conditions.

The **protein concentration** of the salamander glue (in 5% v/v acetic acid) was determined with the Pierce™ Coomassie Plus (Bradford) Assay Kit (Co. Thermo Fischer, USA) according to the supplier’s instructions, with BSA as a calibrant on a nanophotometer (Co. Implen, Germany). Furthermore, 2-mercaptoethanol was added, at a total of 3% of the volume, and heated at 99 °C for 4 min. To change the acidic milieu to an alkaline one, rehydration buffer (7 M urea, 2 M thiourea, 2% CHAPS, 0.002% bromophenol blue) was added as well as 2-mercaptoethanol, again heated three times at 99 °C for 4 min, resulting in a complete dissolution of the sample. All samples, standards and blanks were prepared and measured in triplicate and the absorption was measured at 595 nm.

Quantification of the **carbohydrate concentration** in the salamander secretion was performed by Andrew Smith from Ithaca College, USA, using the orcinol-sulfuric acid method described in protocol 1 by^58^ with modification as described in^40^. In the present study, a 96-well plate approach was used, allowing the quantification of small sample amounts. Three samples (in each case around 1–2 mg), taken from several salamanders were solubilized in concentrated sulfuric acid for 1 hour and heated for 10 minutes at 80 °C. As a reference, different carbohydrates were tested^44^ and the protein bovine serum albumin was used as a negative control.

For the **element analysis** with energy dispersive X-ray spectroscopy (EDX), the freshly collected adhesive was directly attached to blank aluminium stubs and not sputtered with gold or carbon. The sample was directly measured with a Jeol IT 300 (Co. Jeol, Germany) equipped with an EDX spectrometer with a lithium-drifted silicon detector crystal. For evaluation, the software program EDAX Genesis 5.11 (Co. Mahwah, USA) was used. The collecting time of the elements in the samples was 100 s with a ~30% dead time at 20 KeV.

For **element screening analysis** with inductively coupled plasma mass spectrometry (ICP-MS), dried adhesive was homogenized with an agate mortar, digested with (65%) nitric acid (Suprapur) at a heating plate (170 °C) and measured with ICP-MS (model: PerkinElmer Sciex 6100) at Seibersdorf Labor GmbH (Austria). The analysis was performed for alkali metals Li, Na, K and Rb, the alkaline earth metals Be, Mg, Ca, Sr and Ba, and the additional selected elements Fe, As, Al, V, Cr, Mn, Co, Ni, Cu, Zn, Mo and Cd.

The **amino acid composition** of the adhesive secretions was determined by the Institute Kuhlmann (Germany), as described in ref. 37. In brief, tryptophan was determined after alkaline hydrolysis of the sample material with an amino acid analyser. For the determination of cysteine and methionine, the sample material was hydrolysed after a 15-hour performic acid treatment for 24 hours with 6 N HCl at 120 °C and afterwards determined with an amino acid analyser. The remaining amino acids were determined after the acid hydrolysis of the sample material for 24 hours with 6 N HCl at 120 °C, likewise with an amino acid analyser.

The measurements with **ATR-IR spectroscopy** were performed on a Bruker Equinox 55 instrument (Co. Bruker, Germany) with a Harrick Golden Gate ATR inlet (diamond crystal, one reflection). Measurements were taken with a resolution of 4 cm^−1^ and 32 scans. Background measurements were taken against air and water. The **FT-Raman** measurements were performed on a Bruker Vertex 80 with a RAM II module (Co. Bruker, Germany). A 1064 nm Nd Yg laser was used^37^. Measurements were taken with 250 scans at a resolution of 4 cm^−1^. For the analyses, freshly isolated salamander secretions were taken, applied directly to the diamond crystal (ATR-IR) or a gold sputtered slide (FT-Raman) and measured again. Afterwards, the samples were dried for a few hours at room temperature, measured again, and subsequently dried at 80 °C (to completely dry the sample) before being measured a third time.

For the **alkaloid analysis** one individual was anesthetized in 3% (v/v) MS-22, immediately decapitated and directly immersed in methanol solution. The presence of alkaloids was examined using an acid-base alkaloid fractionation, as outlined earlier^59, 60^. The entire skin of one individual *P.* *shermani* was placed in approx. 10 mL of 100% methanol. Gas chromatography-mass spectrometry (GC-MS) was performed of the skin extract on a Varian Saturn 2100 T ion trap MS instrument, which was coupled to a Varian 3900 GC with a 30 m × 0.25 mm i.d. Varian Factor Four VF-5 ms fused silica column. GC separation of compounds was attained using a temperature program from 100 to 280 °C at a rate of 10 °C per minute with helium as the carrier gas (1 mL/min). The fraction was analysed with both electron impact MS and chemical ionization (CI) MS with methanol as the CI reagent.

On the basis of ref. 37, the dried adhesive on Aclar® foil was prepared for gel electrophoreses as follows: Aclar® foil (these samples are referred to as being from Pos 1 and Pos 2) containing glue from one animal was cut from two different positions (approx. 0.75 cm² each), chopped into smaller pieces and placed in two sample vials. To exclude that material from the native foil was eluted, empty foil pieces were used as control. 100 μL of 2x NuPAGE Lithium Dodecyl Sulfate (LDS) Sample Buffer (Co. Life Technologies, USA, Cat-No. NP0007) were added, followed by sonication (5 min) and heating (70 °C, 10 min). 100 μL of 100 mM DTT were added, followed again by sonication and heating. For one-dimensional polyacrylamide gel electrophoresis (**1D-PAGE**), the sample was spun and 10 µL of the supernatant was diluted with water (1:1), applied on pre-cast NuPAGE 4–12% Bis-Tris gels (Co. Life Technologies, USA, Cat-No. NP0326BOX) and separated using MOPS SDS Running Buffer (Co. Life Technologies, USA, Cat-No. NP0001). BenchMark Protein Ladder (Co. Life Technologies, USA, Cat-No. 10747-012) was used as molecular weight marker (kDa). Gels were run at a constant voltage (200 V), a starting current of 100–125 mA/gel for 50 min, and an end current of 60–80 mA/gel.

In addition, glue was scratched from approx. 2 cm^2^ of Aclar® foil, put into a sample tube and dissolved in a buffer containing 2 M thiourea, 7 M urea and 2% (w/v) CHAPS (this sample is referred to as “Mix”). The protein concentration was determined as described. For 1D-PAGE, the sample was spun down and an aliquot of the supernatant was combined with 4x LDS and water to reach a final concentration of max. 10 mg protein per µL in 1x LDS buffer. The sample was separated on 4–12% Bis-Tris gels with a MOPS SDS buffer, as described above.

For two-dimensional polyacrylamide gel electrophoresis (**2D-PAGE**), the dried adhesive was scratched from the Aclar® foil and dissolved in IEF rehydration buffer (2 M thiourea, 7 M urea, 2% (w/v) CHAPS, 0.002% bromophenol blue, 2% (w/v) IPG buffer pH 3–10) overnight. Ready-made IPG strips (7 cm, Immobiline DryStrip, pH 3-10NL; Co. GE Healthcare, UK) were rehydrated with protein-containing rehydration buffer overnight (44 µg) and the sample was separated with a Multiphore II (Co. GE Healthcare, UK) for 45 hours (30 kVh). The strip was equilibrated in 75 mM Tris-HCl pH 8.8 containing 6 M urea, 30% glycerol, 2% SDS, 1% (w/v) DTT and 0.002% bromophenol blue, followed by equilibration in 50 mM Tris-HCl pH 8.8 containing 6 M urea, 30% glycerol, 2% SDS and 4% (w/v) idoacetamide, 15 min each. The second dimension was again run on 4–12% Bis-Tris gels using a MOPS SDS buffer (for details see 1D-PAGE). Proteins were visualized by silver staining according to ref. 61.

In-gel tryptic digestion was carried out as previously described^62^ with the modification that purified peptides were prepared for MALDI-TOF/TOF-MS. For this, the purified tryptic peptides were dissolved in 2 µL matrix solution (4 mg/mL α-Cyano-4-hydroxy cinnamic acid) and applied to stainless steel targets for analysis. **Peptide Mass Fingerprinting** (PMF) and **peptide sequencing** (MS/MS) were carried out in positive ion, reflectron mode on an UltrafleXtreme (Co. Bruker, Germany). For all enzymatic digestion data, autolytic tryptic products, keratin and gel blank artefacts were assigned and removed prior to a database search on an in-house Mascot server v2.4.0^63^. Against a database containing only protein sequences relevant for “amphibian” (NCBInr, 329.171 sequence entries; Aug 31^st^, 2016) and against the latest NCBInr and SwissProt databases (both Aug 2016), both restricted to “chordate”. The database search was performed with the following parameters: monoisotopic mass values, peptide mass tolerance ± 0.3 Da, fragment ion tolerance ± 0.5 Da in the case of MS/MS analysis, one missed cleavage, carboxyamidomethylation as fixed modification and methionine oxidations as variable modification. In the case of an unsuccessful database search, *de novo* sequencing was performed with Biotools v3.2 (Co. Bruker, Germany) using information from the immonium ion region (m/z 40–170) for present amino acids. Amino acid sequences of high statistical probability were searched with BLAST against NCBInr (limited to “amphibia” or *Plethodon*). The search results were listed as a taxonomy report and the most likely results from salamanders were considered as potential protein identification. If more than one peptide from a gel spot gave the same BLAST result, this identification was considered to be an even more correct identification. The supplementary material lists all the BLAST results, while the manuscript discusses only the most likely ones in detail.

### Ethical statement

Salamanders were wild-born individuals collected from populations in the vicinity of Highlands Biological Station, Highlands, North Carolina, USA (collecting permit, Highlands Biological Station) and according to the requirements of local nature conservation authorities. All methods in this study were approved to U.D. by the Institutional Animal Care and Use Committee in the University of Bremen Germany. Only for the alkaloid analyses one individual was anesthetized in 3% (v/v) MS-22, immediately decapitated and directly immersed in methanol solution. Within this study, no experiments with live animal were carried out. Only an absolute minimum of animals necessary to the research were stressed and its glue collected and used for the planned investigations.

## Electronic supplementary material

Supplementary Information
